# Psychometric properties and modification of the 15-item geriatric depression scale among Chinese oldest-old and centenarians: a mixed-methods study

**DOI:** 10.1186/s12877-022-02833-x

**Published:** 2022-02-19

**Authors:** Chi Zhang, Hao Zhang, Minghao Zhao, Chen Chen, Zhongquan Li, Deping Liu, Yali Zhao, Yao Yao

**Affiliations:** 1 Department of Education, Beijing Hospital, National Center of Gerontology, Chinese Academy of Medical Sciences, Beijing, China; 2 Department of Healthcare Policy and Research, Weill Cornell Medical College, Ithaca, NY USA; 3 School of Basic Medicine, Peking University Health Science Center, Beijing, China; 4 Department of Global Health, School of Public Health, Wuhan University, Wuhan, Hubei China; 5 School of Social and Behavioral Sciences, Nanjing University, Nanjing, Jiangsu China; 6 Central Laboratory, Hainan Hospital of Chinese PLA General Hospital, Sanya, Hainan China; 7 China Center for Health Development Studies, Peking University, Haidian District, Beijing, China

**Keywords:** 15-item Geriatric Depression Scale, Oldest-old, Centenarians, Validity

## Abstract

**Background:**

The 15-item Geriatric Depression Scale (GDS-15) shows good performance in detecting depression among older persons, but its applicability has not been well studied in non-Western oldest-old adults and centenarians. This study aimed to evaluate the psychometric property of the GDS-15 and a simplified version among a large representative longevous population in China.

**Methods:**

A total of 1624 individuals (786 oldest-old persons aged from 80 to 99 years; 838 centenarians aged 100+ years) participated in this study. Home interviews with structured questionnaires were conducted to collect sociodemographic data. Depressive symptoms were measured using the Chinese GDS-15 version. We implemented mixed methods for the psychometric evaluation of the GDS-15. Cronbach’s α coefficient and item-total correlation coefficients were used to evaluate the internal consistency. A standard expert consultation was conducted to test the content validity of each item. Multiple factor analyses were used to explore the optimal factor structure and measurement invariance.

**Results:**

The α coefficient of the GDS-15 was 0.745, while two items impaired the overall consistency reliability. Nineteen experts rated the applicability for each item and provided removal suggestion. Five items with less validity were removed, and a simplified 10-item GDS model with three-factor structure was proposed as an optimal solution. The GDS-10 model showed factorial equivalence across age, sex, residence, and education in multi-group confirmatory factor analyses.

**Conclusions:**

The original GDS-15 has acceptable internal reliability, known-group validity, and concurrent validity among Chinese community-dwelling oldest-old and centenarians; however we provided preliminary evidence indicating that individual items related to somatic function or social activities may not be applicable for this population. The modified GDS-10 can be proposed as a potentially more practical and comprehensible instrument for depression screening.

**Supplementary Information:**

The online version contains supplementary material available at 10.1186/s12877-022-02833-x.

## Background

Depression is one of the most common mental health disorders in later life [1]. Early detection of depression is essential in geriatric care due to its increasing prevalence and detrimental effects among the older people worldwide [2, 3]. Older adults with depression or depressive symptoms face with numerous adverse health outcomes including functional decline, cognitive impairment, decreased quality of life [4, 5]. Previous studies have showed that depressive symptoms were more prevalent in oldest-old than in younger old groups [2, 6]. The decline of function associated with aging is closely related to the psychological symptoms of depression in the oldest-old [7]. In addition, social support status is a more important predictor of depression among older people from different cultures compared to the general population [8]. Hence, the symptoms and etiology of depression in late life may be more heterogeneous than in younger people [9]. Oldest-old adults, including centenarians, have constituted the fastest growing segments of the world population [10]. According to China’s General Program for Sustainable Development, China was projected to becoming a super-aged society by 2033 with a life expectancy of over 80 years, and having the largest population of the oldest-old across the globe [11]. However, due to the difficulties in taking a representative sample of the oldest-old and the shortage of psychiatrists, accurate depression screening among this population have not received enough attention [12].

The 15-item Geriatrics Depression Scale (GDS-15) has been widely used for depression screening and has been translated into multiple languages [13]. The GDS-15 was a simplified version of the 30-item long form GDS version developed by Sheik and Yesavage in 1986 [14]. Both ICD-10 criteria and DSM-IV criteria have shown that the GDS-15 was valid for measuring mild and major depression [15, 16]. In a systematic review, the pooled sensitivity, specificity, and area under the ROC curve of the GDS-15 were 79%, 77%, and 0.84 among older adults [9]. In China, however, there are no studies about the GDS-15’s properties in the oldest-old and centenarians; therefore, its efficacy in this population is unclear, and more psychometric evidence is needed. Since older people in an advanced age have cognitive difficulties, a simple yes/no response format in the GDS was more convenient than other measurement tools such as the Center for Epidemiologic Studies Depression Scale and the Beck Depression Inventory. Although the GDS-15 is more practical in clinical practice, the time required for answering all the questions is yet another burden for very old people, especially centenarians. Individual items regarding physical function and social activities are confounded with physical illness symptoms and may be burdensome for frail subjects [17]. It is still unclear whether all 15 items are suitable for Chinese oldest-old and centenarians, who tend to have declining physical abilities, low levels of literacy, and less social involvement. Re-evaluation and optimization of the GDS-15 seem necessary for depression screening in the oldest-old. The existing literature covered evidence about incidences and influencing factors of depression in later life [18, 19]. There were also evidence suggesting that the GDS-15 showed various validities among older adults [20, 21], while the psychometric properties of the GDS-15 among the oldest-old population (80+ years) is not yet clear.

The factor structure of the GDS is an important property when examining depression among samples of different ethnic backgrounds. However, as far as we are aware, the factor structure of the Chinese version GDS-15 among the oldest-old has not been well reported. A meta-analysis showed that conflicting GDS-15 structures (from 1 to 4 factors) were related to cross-cultural diversity in the expression of depressive symptoms among older people [13]. Furthermore, the older adults’ age, residence type, and social function may also contribute to the inconsistent results of the GDS. Several studies from the US and Europe countries obtained a two-factor structure regarding positive and negative emotions [22, 23]. While studies in Asia, including China, have shown that the GDS-15 had 3-4 dimensions [24–26]. Zhao et al. revealed that a three-factor model, including life satisfaction, general depressive affect, and withdrawal, fitted the GDS-15 best among Chinese community-dwelling older adults aged 60 to 99 years [24]. Lai et al. obtained a four-factor model including negative mood, positive mood, inferiority, and disinterested in older Chinese aged 55 years and above living in Canadian [27]. Previous studies have simplified the GDS-15 using multiple methodologies such as factor analysis, internal consistency, or item response theory (IRT) [28, 29]. As a result of the internally consistent reliability and expert consultation, Koenig et al. simplified the GDS to an 11-item version and found that it is sensitive and specific in inpatients [17]. Recently, Nahathai et al. used confirmatory factor analysis and IRT to eliminate 9 items from the original GDS-15 that might cause cultural bias and developed a new version that is comparable to the GDS-15 in its ability to detect depression [30]. A study conducted in the US showed that several items (dropped activities and interests, prefer to stay at home, and mind as clear as it used to be, etc.) in the GDS had poor consistency rate with clinical diagnosis among community older adults [31]. Another study from China showed that a 4-item GDS had equivalent sensitivity (57% vs. 60%), specificity (78% vs. 61%), and better accuracy (67% vs. 63%) in a mildly demented Chinese sample whose mean age was 80.87 years when comparing with the 15-item GDS [32]. These studies indicated that the accuracy of the GDS for screening depression in later life was associated with the conciseness of the scales, and even could be improved through removing some items that do not individually distinguish depression well. Ageing process is associated with function decline and social isolation, and it has been documented that identifying specific psychosocial symptoms from depression or other health conditions in the oldest-old was complicated [33]. One study from the United States showed significant age differences in the scores of specific items and dimensions of the GDS between centenarians and the younger old population [6]. Moreover, lifestyle, education, and social connections directly influence the respondent’s expression of depression [34]. Findings from the Georgia Centenarian Study indicated that it might be difficult to distinguish depressive symptoms from physical symptoms caused by advanced age or fatigue, and the authors called for qualitative studies to address this issue [6]. Also, specific compound sentence patterns in the GDS may challenge older people’s understanding, and a previous Italian study reported additional difficulties among centenarians when answering dichotomous GDS questions due to the lower education and sensory impairment [35].

Despite the GDS-15’s properties being studied across several populations, most previous studies involved older people from Western countries [36] or younger old groups (aged 60 or above) [24, 26], while very few studies examined the oldest-old and centenarians with substantial sample sizes. Besides, existing studies relied on measurement methods to modify the GDS, and qualitative evidence on the applicability for each GDS items among the oldest-old is lacking. To address the gap of GDS-15’ utilities in the oldest-old population, we conducted this mixed-methods designed psychometric study to evaluate the reliability the reliability, structure validity, and measurement invariance of the scale using a large sample of Chinese oldest-old and centenarian persons. We also aim to identify the core depressive symptoms within this population and modify the GDS-15 by combining quantitative and qualitative evidence.

## Participants and methods

### Data source

**Fig. 1:**
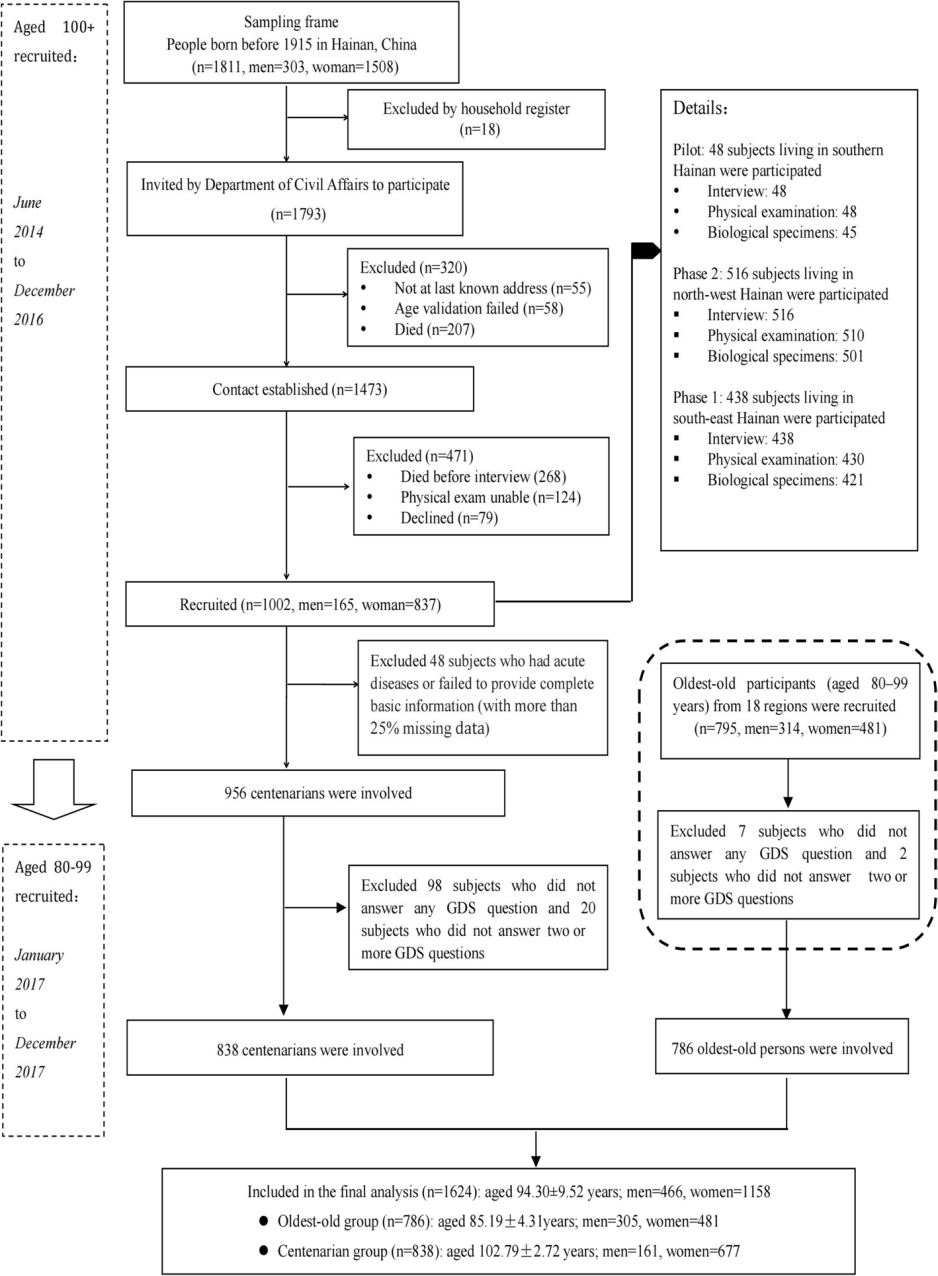
The flowchart of participants recruited and excluded in CHCCS

The data for this study were collected from the China Hainan Centenarian Cohort Study (CHCCS), from June 2014 to December 2017. The CHCCS is a large cohort project designed to assess the physical function, mental health and social status of aging adults, as well as establish indicators for healthy aging [37]. According to the International Expert Committee on Population Aging and Longevity, Hainan Province has the highest percentage of centenarians (18.75/100 000) among all Chinese provinces [38]. Longevous persons live on this island their whole lives; therefore, Hainan province can provide a steady study sample. 1793 centenarians were initially recruited using a complete sampling according to the household registration data provided by the Civil Affairs Bureau method [37], and valid connections were established among 1473 centenarians. Inclusion criteria included: (1) 100 years or older by 1 June 2014; (2) volunteered to participate in the study and provided written informed consent; (3) was conscious and could cooperate to complete the interview and health examinations. 124 subjects who were unable to cooperate due to dementia or paralysis were excluded before the survey. 58 subjects who failed to meet the three-step age verification (Supplementary Figure 1), and 48 participants with more than 25% missing data were also excluded. In the second phase, the oldest-old participants (aged 80–99 years) were recruited as a control group in the second phase from 18 regions in Hainan. In total, 956 centenarians and 795 oldest olds were interviewed at home or health service centres by native nurses who were trained in interviewing older adults and able to speak the local dialect. We further excluded subjects (9 oldest-olds and 118 centenarians) who failed to answer two or more GDS questions. Considering the influence of missing values on the stability of factor analysis, participants with one missing GDS value were addressed using multiple imputation methods. The flowchart of sample selection process of this study was showed in Fig. 1.

**Ethical statement**

 The ethics committee of the Hainan branch of the Chinese People’s Liberation Army General Hospital approved the study protocol (301hn11201601). All participants or their guardians provided written informed consent before participating in the survey.

**Measures**

Depressive symptoms were measured using a Chinese version of the GDS [39]. The scale consists of 15 binary questions in which participants are asked to answer how they felt over the past week (1 = Yes, 0 = No). The total score of the GDS-15 is calculated as the sum of the 15 items, with a higher score indicating more depressive symptoms (possible range 0–15; observed range of 0–15). Participants who were illiterate or had cognitive impairment answered the questions with the help of investigators and their legal representatives. The 10-item Barthel Index was used to measure physical function [40]; subjects were considered exhibiting physical dependence if the total score was 90 points or less [41]. The 7-item Satisfaction With Life Scale (SWLS) was used to assess subjective well-being level (observation range 0–35). Visual Analog Scale (VAS), a 20-cm vertical scale ranging from 0 to 100, was used to record self-rated health status.

**Statistical analysis**

We used mixed methods for the psychometric assessment of the GDS-15. Cronbach’s alpha coefficient (α) and item-total correlation (ITC) were used to evaluate the internal reliability of the GDS-15. We conducted a standard expert consultation by inviting experts who have senior professional titles from the geriatric psychology field in China. Each expert was asked to rate the applicability of each item by a likert-5 score from “not applicable=1” to “very applicable=5”. The expert member panel should also select 3-10 items that can be deleted. As with previous studies that used content validity ratio to shorten scales, when an item in the GDS was selected by more than half of the experts, it was considered a candidate for deletion [42, 43]. Details of the consultation form and experts list were shown in Appendix 1 and 2. Third, Exploratory factor analyses (EFA) were used to explore the optimal factor structure. Retained eigenvalues should meet the K1 criterion (≥ 1) and should be greater than the mean or the 95th percentile of the random samples in the parallel analysis (PA). Items with poor factor loading (<0.5) were considered for removal from the scale [44]. Confirmatory factor analyses (CFA) with robust weighted least squares estimations were performed using Mplus (version 7.4) [45] to compare the fitness of competing GDS models. χ2/df, root mean square error of approximation (RMSEA), comparative fit index (CFI), and normed fit index (NFI) were used to evaluate the fitness. According to criteria recommended by statisticians, a model is considered good (or acceptable) if normed χ2/*df* ≤ 2 (3), RMSEA ≤ 0.06 (0.08), CFI ≥ 0.95 (0.90), and NFI ≥ 0.95 (0.90) [46]. Akaike information criterion (AIC) and Bayesian information criterion (BIC) were also used to evaluate the suitability of default models. Smaller AICs and BICs indicate better fitness for competitive models. Factorial invariance of the GDS across age, sex, residence, and education was tested by multi-group confirmatory factor analyses (MGCFA), which consisted of a series of nested confirmatory steps for parametric constraint models [47]. A non-significant _△_χ^2^ (*P *>0.05), a _△_CFI value<0.01, and a _△_RMSEA value<0.15 between alternative models indicate equivalent fitness of the factor structure across subgroups [48].

## Results

### Demographic characteristics

In total, 1624 individuals (94.30 ± 9.52 years) participated in this study. Among them 786 were oldest-olds (85.19 ± 4.30 years) and 838 were centenarians (102.48 ± 2.74 years). As Table 1 showed, most participants were female (71.3%), Han ethnic (89.4%), illiterate (84.1%), divorced or widowed (70.5%), lived at home (99.4%), and lived in cottages (72.7%). 92.2% of the participants had at least one closely connected relative, while only 42.0% had at least one closely connected friend. The prevalence of physical function dependence was 47.4%. The average summed GDS-15 score was 4.38 ± 3.02 (5.23 ± 3.24 for centenarians and 3.56 ± 2.50 for the oldest-old). Compared to the excluded participants who failed to respond enough GDS questions (*n* = 127), participants who were included in the final analysis were more likely to be younger, male, and lived in rural area (Supplementary Tables 1, *Ps* < 0.05).

**Table 1:** Demographic characteristics and GDS-15 scores of the 1624 participants

Characteristics		GDS score (mean ± SD)					
		Total sample (*N *=1624)		Oldest-old(*N *=786)		Centenarians(*N *=838)	
Age, mean ± SD		94.30 ± 9.52	4.38 ± 3.02	85.19 ± 4.20	3.56 ± 2.50	102.48 ± 2.74	5.23 ± 3.24
Sex, n (%)	Male	466(28.7)	3.48 ± 2.54	305(38.8)	3.13 ± 2.31	161(19.2)	4.17 ± 2.87
	Female	1158(71.3)	4.74 ± 3.11	481(61.2)	3.83 ± 2.58	677(80.8)	5.40 ± 3.32
Education, n (%)	Illiterate	1365(84.1)	4.57 ± 3.09	603(76.7)	3.70 ± 2.59	762(90.9)	5.28 ± 3.31
	Literate	259(15.9)	3.34 ± 2.29	183(23.3)	3.10 ± 2.11	76(9.1)	3.99 ± 2.69
Residence, n (%)	Rural	1057(65.1)	4.53 ± 3.03	481(61.2)	3.71 ± 2.57	576(68.7)	5.23 ± 3.23
	Urban	567(34.9)	4.09 ± 2.95	305(38.8)	3.33 ± 2.37	262(31.3)	5.02 ± 3.37
Ethnicity, n (%)	Han	1452(89.4)	4.32 ± 3.01	712(90.6)	3.52 ± 2.53	740(88.3)	5.12 ± 3.27
	Minority	172(10.6)	4.81 ± 2.96	74(9.4)	3.90 ± 2.25	98(11.7)	5.61 ± 3.36
Marriage status, n (%)	Married	479(29.5)	3.34 ± 2.32	389(49.5)	3.05 ± 2.11	90(10.7)	4.63 ± 2.76
	Divorced or widowed	1145(70.5)	4.82 ± 3.18	397(50.5)	4.07 ± 2.74	748(89.3)	5.23 ± 3.33
Residence type, n (%)	Cottage	1180(72.7)	4.42 ± 3.13	592(75.3)	3.57 ± 2.66	619(73.9)	5.20 ± 3.32
	Building or others	444(27.3)	4.32 ± 2.58	194(24.7)	3.56 ± 1.99	219(26.1)	5.05 ± 2.86
Living situation, n (%)	At home	1615(99.4)	4.46 ± 3.12	785(99.9)	3.71 ± 2.84	830(99.0)	5.25 ± 3.39
	Nursing home	9(0.6)	4.33 ± 3.42	1(0.1)	0	8(1.0)	4.87 ± 3.22
Relatives contacts, n (%)	Yes ^a^	1497(92.2)	4.42 ± 3.01	734(93.4)	3.57 ± 2.52	763(91.1)	5.24 ± 3.22
	NO	127(7.8)	4.44 ± 2.98	52(6.6)	4.20 ± 2.32	75(8.9)	4.56 ± 3.25
Friends contacts, n (%)	Yes ^b^	682(42.0)	4.49 ± 3.15	409(52.0)	3.64 ± 2.53	273(32.6)	5.74 ± 3.52
	NO	942(58.0)	4.29 ± 2.88	377(48.0)	3.44 ± 2.41	565(67.4)	4.88 ± 3.02
Physical function, n (%)	Dependence^ c^	770(47.4)	5.34 ± 3.13	202(25.7)	4.42 ± 2.58	568(67.8)	5.74 ± 3.28
	Independence	854(52.6)	3.51 ± 2.61	584(74.3)	3.32 ± 2.42	270(32.2)	4.02 ± 2.99

*GDS* geriatric depression scale, *SD* standard deviation

^a^ Participants self-reported having at least one closely connected relative, ^b^ Participants self-reported having at least one closely connected friend, ^c^ Participants with a Barthel Index score of 90 or less were defined as physical dependence

**Internal consistency**

The α coefficient of the GDS-15 was 0.745 and increased after either item 9 or item 15 was deleted (Table 2). The item-total correlation coefficient ranged from 0.354 to 0.651 and mean of ITCs was 0.479.

**Table 2:** Internal consistency and content validity of the GDS-15

Item	Content	Total sample (*N *=1624)		Oldest-old sample (*N *=786)		Centenarian sample (*N *=838)		Applicability score ^a^ (*N *=19)		Delete selected ^b^ (*N *=18)
		α	ITC	α	ITC	α	ITC	Mean	SD	
1	Are you generally satisfied with your life?	0.736	0.401	0.704	0.325	0.761	0.396	3.56	1.16	7 (18)
2	Have you given up many of your activities and hobbies?	0.729	0.522	0.680	0.534	0.762	0.439	2.39	1.12	14 (18)
3	Do you find life boring or empty?	0.710	0.651	0.673	0.571	0.736	0.667	4.28	1.00	3 (18)
4	Do you often feel bored?	0.726	0.519	0.683	0.509	0.753	0.506	4.56	0.69	0 (18)
5	Are you in good spirits most of the time?	0.741	0.418	0.701	0.350	0.752	0.528	4.50	1.02	1 (18)
6	Are you afraid something bad is going to happen to you?	0.723	0.556	0.689	0.470	0.748	0.564	3.94	1.20	4 (18)
7	Are you happy most of the time?	0.742	0.410	0.712	0.330	0.751	0.540	4.33	0.89	3 (18)
8	Do you ever feel like no one is helping you?	0.736	0.406	0.695	0.417	0.761	0.383	3.17	1.09	11 (18)
9	Do you prefer to stay at home, rather than going out?	0.750	0.354	0.718	0.331	0.779	0.374	1.94	0.81	17 (18)
10	Do you feel you have more problems with memory than most?	0.734	0.462	0.697	0.425	0.761	0.447	2.89	1.05	10 (18)
11	Do you think it is wonderful to be alive now?	0.727	0.505	0.688	0.471	0.751	0.522	3.61	1.05	5 (18)
12	Do you feel worthless?	0.713	0.637	0.671	0.609	0.743	0.608	4.06	0.93	1 (18)
13	Do you feel full of energy?	0.739	0.422	0.711	0.361	0.758	0.456	3.83	0.97	3 (18)
14	Do you feel that your situation is hopeless?	0.722	0.565	0.682	0.519	0.745	0.593	3.89	1.06	3 (18)
15	Do you think that most people are better off than you are?	0.747	0.354	0.719	0.322	0.766	0.392	2.78	1.06	12 (18)
	GDS-15	0.745	0.479	0.713	0.436	0.776	0.488			

*GDS* geriatric depression scale; α: Cronbach’s alpha coefficient of the *GDS* when one item was deleted, *ITC* item-total correlation coefficient

^a^ Score of applicability for 15 items from 19 experts, ^b^ Selection of the removal of 15 items from 18 experts

**Content validity**

We obtained feedbacks from 19 geriatric psychologists on the applicability of each item. The average working lives of the experts was 24.3 years, and their advisory opinions were summarized in Table 2. Five items scored below 3.5 point for applicability, of which item 9 (1.94 ± 0.81), item 2 (2.39 ± 1.12), and item 15 (2.78 ± 1.06) were the lowest three. Among the 18 experts who provided suggestions on the removal of items, more than 9 experts chose to delete item 9 (17/18), item 2 (14/18), item 15 (12/18), item 8 (11/18), and item 10 (10/18).

**Factor structure**

Kaiser-Meyer-Olkin (0.801) and Bartelt’s sphere tests (χ^2^ = 1258.153, *df*=105, *P *< 0.001) supported the feasibility of the structure detection. In the first phase, we conducted parallel analysis for all 15 items and 4 factors were extracted. As Table 3 showed, the four factors (psychological perception; positive moods; negative moods and individual activities) accounted for 54.29% of the variance. Items with low reliability, poor factor loading, or recommendations for removal from more than 1/3 of the experts would be considered for removal. We also referred to the items that have been deleted in previous studies. Items 2, 9 and 15 had the lowest content validity, and items 9 and 15 impaired the overall consistency of the GDS. Besides, in an IRT study we have previously published, items 2 and 9 showed unacceptable guess parameter (>0.4) which indicated that the respondents might not provide truthful responses when answering these two questions [49]. Therefore, we deleted the above three items, and three factors were extracted from the remaining 12 items. In the GDS-12 model, two items (1 and 8) still showed poor factor loading (<0.5). Considering that more than 1/3 of the experts recommended deleting item 1 and 8, and they have also been suggested to deleted in some previous studies, we further deleted these two items and repeated the parallel analyses. Three factors explaining 60.86% of the variation were extracted and all the 10 items showed good or excellent loadings (>0.6). The three factors in the GDS-10 model were defined as psychological perception (items 2, 4, 11, and 14), positive moods (items 5, 7 and 13), and negative moods (items 6, 10 and 12). Scree plots of three GDS versions were shown in Supplementary Figure 2. The EFAs results remained consistent when excluding 43 participants with one missing GDS value (Supplementary Table 1).

**Table 3:** Factors and item loadings of three Geriatrics Depression Scale models

Model	GDS-15				GDS-12			GDS-10		
Factor	1	2	3	4	1	2	3	1	2	3
Item-1	0.516	0.303	-0.238	0.136	0.437	0.336	-0.136	——	——	——
Item-2	0.058	0.161	0.139	0.625	——	——	——	——	——	——
Item-3	0.634	0.218	0.344	-0.017	0.611	0.205	0.333	**0.742**	0.217	0.215
Item-4	0.734	0.065	0.127	-0.113	0.684	0.157	-0.018	**0.823**	0.058	-0.005
Item-5	0.249	0.695	0.089	0.011	0.197	0.772	0.091	0.165	**0.766**	0.062
Item-6	0.103	0.121	0.760	0.058	0.190	0.115	0.734	0.132	0.105	**0.795**
Item-7	0.374	0.684	-0.038	0.059	0.255	0.774	-0.061	0.286	**0.761**	-0.071
Item-8	0.404	0.060	0.058	0.380	0.433	0.039	0.098	——	——	——
Item-9	-0.055	-0.049	0.053	0.845	——	——	——	——	——	——
Item-10	-0.066	0.045	0.790	0.111	0.029	-0.014	0.782	0.022	0.014	**0.819**
Item-11	0.613	0.475	0.041	0.109	0.573	0.370	0.113	**0.629**	0.396	0.014
Item-12	0.348	0.160	0.634	0.075	0.310	0.052	0.655	0.473	0.125	**0.601**
Item-13	-0.11	0.728	0.221	0.113	-0.166	0.638	0.409	-0.112	**0.716**	0.236
Item-14	0.561	-0.032	0.421	0.113	0.668	0.006	0.256	**0.688**	0.051	0.389
Item-15	0.348	-0.401	0.464	0.138	——	——	——	——	——	——
Eigenvalue	2.425	2.086	2.273	1.362	2.286	2.015	1.977	2.154	2.005	1.927
Variance, %	16.17	13.90	15.15	9.08	19.05	16.79	16.48	21.54	20.05	19.27
	54.29%				52.32%			60.86%		

*GDS* geriatric depression scale

**Model fitness and factorial invariance**

We conducted multi-group confirmatory factor analyses to compare the fitness of GDS models. We included four commonly used models as candidates from previous studies [24, 27, 28, 50, 51], and modified GDS versions with more than half of items removed were not included as most fitness indexes are closely related to item numbers in a scale. As summarized in Table 4, multiple indexes were used to compare the fitness of seven competing GDS models. The GDS-10 model (Model C) from the EFAs fitted the data better than the other models (χ^2^/*df=*1.94, CFI=0.976, RMSEA=0.048), and had an appropriate α coefficient and the highest ITC. Although the Model A, B, and F also had an acceptable CFI (> 0.9), the Model C showed smaller χ^2^/*df*, RMSEA, AIC, and BIC, and could be proposed as an optimal solution. The CFA model of the GDS-10 was shown in Supplementary Figure 3. We tested the factorial equivalence of the GDS-10 model using MGCFA. The configural invariance model (free parameters) was used as a basic model and three restrictive models (restrict loading, intercept, and residual sequentially) were tested in a stepwise manner. Results in Table 5 showed that the metric and scalar models had excellent fitness across age, sex, residence, and education (*P >*0.05, △CFI<0.01, _△_RMSEA<0.15) which indicated sufficient structural comparability between subgroups. According to the significance of _△_χ^2^, the measurement invariance of the residual restricted model was not well supported.

**Table 4:** Comparison of fitness across 7 competing GDS models in Chinese oldest-old and centenarians

Model	Factor/item	Deleted items	χ^2^/*df*	RMSEA	NFI	CFI	Factor loadings average (min, max)	α	MITC	AIC	BIC
A	1/15	——	2.56	0.061	0.863	0.935	0.38(0.07, 0.71)	0.768	0.479	254.619	261.333
B	3/12	2,9	3.16	0.072	0.866	0.903	0.54(0.25, 0.81)	0.778	0.485	242.481	246.272
C^a^	3/10	1,2,8,9,15	1.94	0.048	0.954	0.976	0.60(0.41, 0.84)	0.767	0.515	170.762	173.317
D	2/15	——	3.63	0.079	0.785	0.831	0.43(0.16, 0.68)	0.768	0.479	275.246	277.597
E	4/15	——	4.23	0.088	0.753	0.795	0.48(0.19, 0.77)	0.768	0.479	294.247	297.124
F	2/11	2,8,9	3.76	0.081	0.871	0.901	0.51(0.27, 0.67)	0.765	0.492	260.124	264.165
G	3/15	——	4.53	0.092	0.718	0.762	0.47(0.20, 0.64)	0.768	0.479	301.242	303.759

*GDS* geriatric depression scale, *RMSEA* root men square error of approximation, *NFI* normed fit index, *CFI* comparative fit index, *MITC* mean of item-total correlation coefficient, *AIC* Akaike information criterion, *BIC* Bayesian information criterion

Model A: one factor with 15 items

Model B: factor 1 (with items 1, 3, 4, 8, 11, 14); factor 2 (with items 5, 7, 13); factor 3 (with item 6, 10, 12)

Model C: factor 1 (with items 3, 4, 11, 14); factor 2 (with items 5, 7, 13); factor 3 (with items 6, 10, 12)

Model D: factor 1 (with items 1, 3, 4, 5, 6, 7, 8, 11, 12, 14, 15); factor 2 (with items 2, 9, 13)

Model E: factor 1 (with items 3, 4, 8, 11, 14, 15); factor 2 (with items 1, 5, 7); factor 3 (with items 2, 10, 12, 13); factor 4 (with items 6, 9)

Model F: factor 1 (with items 1, 5, 7, 11); factor 2 (with items 3, 4, 6, 10, 12, 14, 15)

Model G: factor 1 (with items 2, 3, 4, 6, 8, 12, 14, 15); factor 2 (with items 1, 5, 7, 11); factor 3 (with items 9, 10, 13)

^a^ The GDS-10 model derived from the exploratory factor analysis

**Table 5:** Factorial invariance of the GDS-10 model across age, sex, residence, and education

Subgroups	Models	χ^2^/*df*	CFI	NFI	RMSEA	Δχ^2^	*P*	ΔCFI	ΔNFI	ΔRMSEA
Age										
Oldest-old		2.232	0.954	0.977	0.049					
Centenarians		1.813	0.955	0.976	0.041					
	Configural	2.248	0.987	0.977	0.028	——	——	——	——	——
	Metric	2.435	0.972	0.97	0.030	5.343	0.456	0.001	0.005	-0.001
	Scalar	4.111	0.956	0.966	0.044	8.892	0.187	-0.006	0.001	0
	Residual	5.893	0.933	0.943	0.056	25.132	<0.001	0.002	0.003	0
Sex										
Male		2.452	0.944	0.955	0.061					
Female		1.336	0.964	0.978	0.041					
	Configural	2.558	0.955	0.946	0.055	——	——	——	——	——
	Metric	2.765	0.945	0.935	0.046	7.212	0.325	0	-0.001	0.002
	Scalar	4.211	0.951	0.945	0.044	11.456	0.254	0.001	0.001	-0.001
	Residual	6.009	0.954	0.955	0.053	28.462	<0.001	0.009	-0.005	-0.004
Residence										
Rural		1.467	0.961	0.974	0.038					
Urban		2.554	0.948	0.987	0.045					
	Configural	2.758	0.956	0.977	0.042	——	——	——	——	——
	Metric	3.165	0.955	0.977	0.041	8.049	0.289	-0.001	-0.002	0.002
	Scalar	4.311	0.953	0.975	0.038	13.551	0.116	0.003	-0.001	0.001
	Residual	6.109	0.961	0.976	0.042	51.402	<0.001	0.006	0.002	0.001
Education										
Illiterate		1.502	0.965	0.960	0.035					
Literate		2.876	0.969	0.941	0.046					
	Configural	4.189	0.943	0.969	0.045	——	——	——	——	——
	Metric	4.232	0.946	0.965	0.045	4.604	0.614	0.009	0.001	0
	Scalar	4.503	0.934	0.961	0.047	5.951	0.232	-0.002	0.002	-0.002
	Residual	5.679	0.914	0.915	0.054	19.244	0.016	0.006	0.004	0.003

*GDS* geriatric depression scale, *RMSEA* root men square error of approximation, *NFI* normed fit index, *CFI* comparative fit index

**Concurrent validity**

The mean ADL, SWLS, and VAS score was 83.63 ± 22.45, 21.98 ± 6.59, and 61.92 ± 15.26, respectively. The GDS-15 summed score was significantly negatively correlated with ADL (*r *=-0.310, *P *<0.001), SRH (*r *=-0.424, *P *<0.001) and SWLS (*r *=-0.273, *P *<0.001). Consistently, significant correlations were also found among the simplified GDS-10 with theoretically relevant health outcomes (*r *= -0.302 for ADL, -0.415 for SRH, -0.323 for SWLS).

## **Discussion**

This study evaluated the internal consistency reliability, content validity, concurrent validity, and factor structure of the GDS-15 among Chinese oldest-old and centenarians. We also provided valuable suggestion for measuring depressive symptoms among this population and a simplified 10-item GDS version was proposed.

The acceptable internal consistency (α = 0.745) of the GDS-15 in our study was consistent with previous studies from China [27, 52] and other countries [28, 53]. We found that the overall α coefficient increased when deleting item 9 (Do you prefer to stay at home, rather than going out?) or item 15 (Do you think that most people are better off than you are?). Similarly, a study showed poor item-total correlation of item 2, 9, and 15 with the summed GDS-15 score among American community-based older adults [31]. Another study also reported that the GDS’s α coefficient increased when deleting the item 2 and 9 using a sample of older residents in Iran [28]. Unacceptable guessing parameters of items 2 (Have you given up many of your activities and hobbies?) and item 9 found in our published IRT study indicated that subjects without depressive symptoms would also respond to these two questions by guessing [49]. Previous IRT studies also showed that items 1, 2, 9, and 15 had significant differential item function between age and sex [54, 55]. In addition, items 2, 9, and 15 were the three most frequently deleted questions in our expert consultation approach due to lower content validity ratio. In the current study, depressive symptoms were negatively associated with physical function, life satisfaction, and self-reported health. Both the GDS-15 and the 10-item simplified version were found to have appropriate concurrent validity. The shorter version of the GDS showed potential predictive value for quality of life outcomes among older adults.

Longevous individuals in Hainan followed a specific lifestyle due to their advanced age and culture. Items 2 and 9 were related to the subject’s somatic ability, while older adults in Hainan had a higher prevalence of physical dependence (47.4%). Item 15 measures social communication, but the community-dwelling oldest-old and centenarians showed more social isolation compared with those living in cities or long-term care facilities. Most of the participants in the current study were divorced or widowed (70.5%), lived in rural areas (65.1%) and sparse cottages (72.7%), and had no closely connected friend (58.0%). Thus, the above three items might impair the overall reliability and we deleted them in the EFAs. Besides, item 9 was considered to exhibit a prominent cultural bias related with lifestyles of older persons, and several researches have recommended that this item be removed from the GDS [30]. In addition, since the original Chinese GDS-15 version was translated by researchers in Hong Kong, its wording may not be fully applicable to older people in mainland China. Also, the three items are compound statements rather than single sentences which may cause confusion due to the subjects’ high illiteracy rate (84.1%).

Item 1 and 8 were further deleted in consideration of insufficient factor loadings as well as expert consultation. As psychometricians suggested, satisfaction and depression could be considered as two independent latent traits, and item 1 is a general indicator of life satisfaction rather than a unique indicator of depression. Sheikh and colleagues also found that “satisfaction” did not load on any of the factors [56]. Item 8 (Do you ever feel like no one is helping you?) can be regarded an indicator of losing control of mental wellbeing as well as social avoidance. Although it might be a powerful indicator of depression from a clinical point of view, we need take the subjects’ living conditions into account. The community-based oldest-old in Hainan, especially centenarians, were more socially isolated than those living in nursing institutions, and item 8 might not be a typical depression indicator as well as item 15. Despite the potential instability of factor analyses, this psychometric method has been widely used in most validity studies. Tang and colleagues obtained a stable and comparable GDS models in both Chinese rural and urban samples by deleting four items with poor loadings [50]. In a few studies using EFA, poor loadings of these deleted items were also found. A study including Chinese immigrants aged 55+ years in Canada showed that factor loadings of item 1 and 2 were lower than 0.45 [27]. Poor loadings of item 8, 9 and 15 were also found in three community-based studies in Japan [25, 57] and New York [23]. Although the five deleted items have also been shown to be inappropriate in several previous studies, inconsistent results also existed. A study conducted by Daniel et al. showed good loadings in four factors for all the 15 items in urban Chinese older adults [26]. Unlike in Hainan, participants in Daniel’s study were younger, had higher education level, and living in crowded residential buildings. A well fitted 3-factor model with all loadings above 0.5 was also found in another study conducted among general older adults in Mainland China [24].

Studies assessing the construct structure of the GDS-15 have largely mixed findings which may be associated with culture, language, and sample heterogeneity [13, 27]. The four factors structure of the GDS-15 obtained in this study was found in studies from Japanese [57], Greek [58] and China [26]. However, two studies from Columbia and New York and have shown that the GDS-15 had a two factors structure including positive and negative moods [23, 59]. In contrast, studies in Asia generally found that the GDS-15 has 3-4 dimensions. Cultural diversities are one of the main reasons for these mixed results. The older persons in Western countries dare to directly express their emotional feeling to the people around them, while Chinese older people are more bashful. After five less valid items were deleted, the revised GDS-10 model showed better fitness than competing models (Table 4). Depression symptoms in Chinese oldest-old could be defined as a multidimensional concept including psychological perception (4 items), positive moods (3 items), and negative moods (3 items). Positive and negative moods can be considered two common depression dimensions [60], which have been examined in studies from Turkey [61], Korea [62], US [62], and China [27]. Although previous studies have confirmed the equivalence of the long-(30 items) and short-(15 items) form GDS for both sexes [24, 63], few studies have reported its equivalence across age groups, and especially for centenarians. Our MGCFA results confirmed the factorial invariance of the revised GDS-10 model, which indicated that the patterns of the three-factor model were equivalent across age, sex, education, and residence subgroups. For instance, despite concerns that demographic differences exist between the oldest-old and centenarians, the age invariance indicated that subjects across the two subgroups responded to the scale with the same underlying framework. Besides, the cross-educational equivalence of the GDS-10 supported its stable validity for illiterate oldest-old.

We matched several modified GDS versions with our GDS-10 and found that item combinations involved in different well performed simplified GDS versions was closely associated with the culture, age, and life condition of the older people. For example, items 1, 8, 9, 15 were deleted from four GDS versions (3-6 items) used in Turkey [61], whereas two 5-item GDS versions widely used in European and American contained 4 items that were deleted in our study [64, 65]. Similarly, when younger older people (>60 years) were screened for depression, some fatigue symptoms (such as item 2 and 9) were involved in a 12-item Chinese GDS version developed by Xie and colleagues using a Delphi method [66]. In Kathryn’s study [31], items 2 and 9 had poor accuracy for American older people (82.3 years) from nursing homes, while items 8 and 15 were of high accuracy. These results showed that physical function symptoms were not appropriate for the oldest-old while the applicability of social symptoms were associated with residence styles of the subject. In general, social activities related items were more often involved in settings conducted in long-term care services than in communities.

One strength of this study is the considerable sample size of oldest-old and centenarian adults from a non-Western country. Another strength is that we identified potential typical depression indicators in this special population using a mixed-methods approach of measurement proprieties and expert-based panel evaluation. Multiple aspects of the modified 10-item GDS version confirmed in the current study would provide quantitative and qualitative psychometric evidences for accurate depression screening among the oldest-old population. The study further suggested that in addition to emotional factors, physical function and social support status of the subjects should also be considered in depression screening, which is also applicable to other relevant studies. Several limitations should be noted. First, we were unable to conduct clinical depression diagnosis during the 3 years extensive survey due to the community-dwelling design of the CHCCS, thus sensitivity or specificity analyses were lacking. Further studies including standard clinical diagnostic procedures are warranted to test the accuracy of different GDS versions. However, 7 items in our simplified GDS-10 were included in the DSM-5 golden standard which might support the scale’s screening performance. Second, we did not include cognitive impairment as one of the exclusion criteria, as some previous studies have done [57, 67]. In the initial sample, we excluded participants were unable to establish a valid connection due to dementia or palsy (Fig. 1). Thus, we were able to ensure that subjects included in the final analysis could answer the GDS questions. In addition, the face-to-face interview conducted by a professional medical team including neurologists could reduce the difficulties in understanding and answering GDS questions. Third, although the sample of this study included a large number of community-based oldest-old adults, the subjects were all exclusively from one province, and generalization of the findings to older people from long-term care services should be done with caution. Fourth, since the option to add or replace items was not presented in the expert consultation form, we might have missed potentially valuable depression indictors when revising the GDS.

## Conclusions

The GDS-15 has acceptable properties among Chinese oldest-old adults and centenarians. From the perspective of psychometric assessment, emotional symptoms are potential typical depression indicators for Chinese community-dwelling oldest-old, rather than those related to somatic function and social activity. The modified 10-item GDS with three factors could be proposed as a more practical and comprehensible instrument for depression screening among this population.

## Supplementary Information

**Additional file 1:** **Figure S1**. Three-steps age validation process of centenarians in CHCCS. **Figure S2**. Scree plots of the threeGeriatrics Depression Scale versions in parallel analysis. Eigenvalues >1and greater than the corresponding eigenvalue from the random data (either theaverage or the 95th percentile) were retained. **Figure S3**. Three-factor GDS-10 model for Chinese oldest-old andcentenarians. Factor 1: psychologicalperception (item 3, 4, 11 and 14); Factor 2: positive moods (item 5, 7 and 13);Factor 3: negative moods (item 6, 10 and 12). **Table S1**. Demographic characteristics of the finally analysed sample and excludedparticipants. **Table S2**. Factors analyses of threeGDS versions among 1581 participants without missing value. **Appendix 1. **Consultation form of the applicabilityof the GDS-15. **Appendix 2. **Information of 19 experts inthe consultation

## Data Availability

The datasets generated for this study are available on request to the corresponding author.
