# Supplementary material for: Psychometric properties and modification of the 15-item geriatric depression scale among Chinese oldest-old and centenarians: a mixed-methods study
Source: BMC Geriatr. 2022 Feb 19;22:144. doi: 10.1186/s12877-022-02833-x (PMC8858470; doi:10.1186/s12877-022-02833-x)
Supplement: Supplementary file 1 — Additional file 1: Figure S1. Three-steps age validation process of centenarians in CHCCS. Figure S2. Scree plots of the threeGeriatrics Depression Scale versions in parallel analysis. Eigenvalues >1and greater than the corresponding eigenvalue from the random data (either theaverage or the 95th percentile) were retained. Figure S3. Three-factor GDS-10 model for Chinese oldest-old andcentenarians. Factor 1: psychologicalperception (item 3, 4, 11 and 14); Factor 2: positive moods (item 5, 7 and 13);Factor 3: negative moods (item 6, 10 and 12). Table S1. Demographic characteristics of the finally analysed sample and excludedparticipants. Table S2. Factors analyses of threeGDS versions among 1581 participants without missing value. Appendix 1. Consultation form of the applicabilityof the GDS-15. Appendix 2. Information of 19 experts inthe consultation [file 12877_2022_2833_MOESM1_ESM.doc]

**Abbreviations**

CFA: confirmatory factor analysis; CFI: comparative fit index; CHCCS: China Hainan Centenarian Cohort Study; EFA: exploratory factor analysis; GDS geriatric depression scale; ITC: item-total correlation coefficient; MITC: mean of item-total correlation coefficient; MGCFA: multi-group confirmatory factor analyses; NFI: normed fit index; PA: parallel analysis; RMSEA: root men square error of approximation.

**Supplementary Information**

**Supplementary Figure 1**. Three-steps age validation process of centenarians in CHCCS. **Supplementary Figure 2**. Scree plots of the three Geriatrics Depression Scale versions in parallel analysis. Eigenvalues >1 and greater than the corresponding eigenvalue from the random data (either the average or the 95th percentile) were retained. **Supplementary Figure 3**. Three-factor GDS-10 model for Chinese oldest-old and centenarians. Factor 1: psychological perception (item 3, 4, 11 and 14); Factor 2: positive moods (item 5, 7 and 13); Factor 3: negative moods (item 6, 10 and 12). **Supplementary Table 1**. Demographic characteristics of the finally analysed sample and excluded participants. **Supplementary Table 2**. Factors analyses of three GDS versions among 1581 participants without missing value. **Appendix 1.** Consultation form of the applicability of the GDS-15. **Appendix 2.** Information of 19 experts in the consultation.


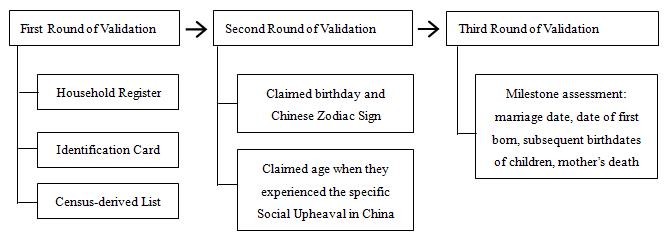


**Supplementary Figure 1. Three-steps age validation process of centenarians in CHCCS.**


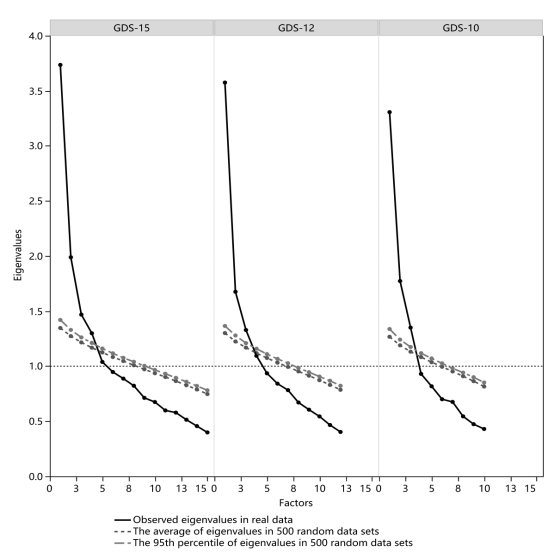


**Supplementary Figure 2.** Scree plots of the three Geriatrics Depression Scale versions in parallel analysis. Eigenvalues >1 and greater than the corresponding eigenvalue from the random data (either the average or the 95th percentile) were retained.


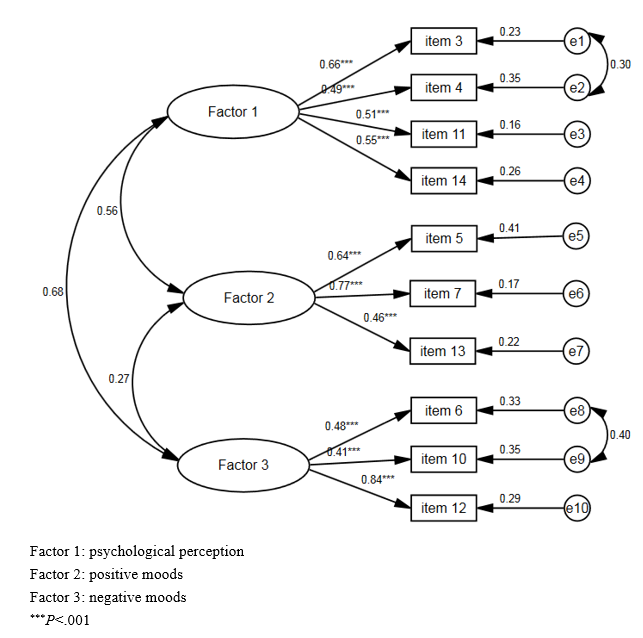


**Supplementary Figure 3.** Three-factor GDS-10 model for Chinese oldest-old and centenarians. Factor 1: psychological perception (item 3, 4, 11 and 14); Factor 2: positive moods (item 5, 7 and 13); Factor 3: negative moods (item 6, 10 and 12).

**Supplementary Table 1. Demographic characteristics of the finally analysed sample and excluded participants**

| Characteristics | | Total (N=1751) | Included  (N=1624) | Excluded  (N=127) | t/χ^2^ | *P*-value |
| --- | --- | --- | --- | --- | --- | --- |
| Age, mean ± SD | | 94.80±9.45 | 94.28±9.51 | 101.49±5.29 | 13.71 | <0.001 |
| Sex, n (%) | Male | 490(28.0) | 466(28.7) | 24(18.9) | 5.89 | 0.015 |
|  | Female | 1261(72.0) | 1158(71.3) | 103(81.1) |  |  |
| Education, n (%) | Illiterate | 1479(84.5) | 1365(84.1) | 114(89.8) | 2.98 | 0.084 |
|  | Literate | 272(15.5) | 259(15.9) | 13(10.2) |  |  |
| Residence, n (%) | Rural | 1107(63.2) | 1057(65.1) | 50(39.4) | 32.06 | <0.001 |
|  | Urban | 644(36.8) | 567(34.9) | 77(60.6) |  |  |
| Ethnicity, n (%) | Han | 1560(89.1) | 1452(89.4) | 108(85.0) | 2.25 | 0.133 |
|  | Minority | 191(10.9) | 172(10.6) | 19(15.0) |  |  |

**Supplementary Table 2. Factors analyses of three GDS versions among 1581 participants without missing value**

| Model | GDS-15 | | | |  | GDS-12 | | |  | GDS-10 | | |
| --- | --- | --- | --- | --- | --- | --- | --- | --- | --- | --- | --- | --- |
| Factor | 1 | 2 | 3 | 4 |  | 1 | 2 | 3 |  | 1 | 2 | 3 |
| Item-1 | 0.527 | 0.148 | -0.069 | 0.109 |  | 0.403 | 0.134 | -0.081 |  | —— | —— | —— |
| Item-2 | 0.080 | 0.288 | 0.361 | 0.517 |  | —— | —— | —— |  | —— | —— | —— |
| Item-3 | 0.618 | 0.160 | 0.352 | 0.033 |  | 0.658 | 0.150 | 0.292 |  | 0.763 | 0.155 | 0.195 |
| Item-4 | 0.672 | 0.095 | 0.174 | -0.160 |  | 0.718 | 0.073 | 0.067 |  | 0.811 | 0.082 | -0.035 |
| Item-5 | 0.237 | 0.705 | 0.002 | -0.149 |  | 0.178 | 0.766 | -0.059 |  | 0.161 | 0.768 | -0.079 |
| Item-6 | 0.094 | 0.047 | 0.799 | 0.027 |  | 0.120 | 0.046 | 0.787 |  | 0.147 | 0.048 | 0.792 |
| Item-7 | 0.294 | 0.723 | -0.077 | -0.128 |  | 0.242 | 0.763 | -0.124 |  | 0.185 | 0.783 | -0.132 |
| Item-8 | 0.452 | -0.033 | 0.033 | 0.167 |  | 0.410 | 0.029 | 0.127 |  | —— | —— | —— |
| Item-9 | 0.170 | -0.194 | 0.059 | 0.831 |  | —— | —— | —— |  | —— | —— | —— |
| Item-10 | -0.013 | -0.061 | 0.781 | 0.066 |  | 0.006 | -0.063 | 0.802 |  | 0.039 | -0.062 | 0.811 |
| Item-11 | 0.532 | 0.304 | 0.078 | 0.093 |  | 0.590 | 0.347 | 0.14 |  | 0.630 | 0.381 | 0.154 |
| Item-12 | 0.359 | 0.104 | 0.608 | 0.170 |  | 0.380 | 0.102 | 0.657 |  | 0.424 | 0.103 | 0.632 |
| Item-13 | -0.021 | 0.714 | 0.122 | 0.314 |  | -0.051 | 0.732 | 0.206 |  | -0.064 | 0.715 | 0.211 |
| Item-14 | 0.539 | -0.060 | 0.404 | 0.078 |  | 0.524 | -0.004 | 0.420 |  | 0.657 | 0.026 | 0.378 |
| Item-15 | 0.220 | -0.423 | 0.459 | 0.131 |  | —— | —— | —— |  | —— | —— | —— |
| Eigenvalue | 2.409 | 2.313 | 2.000 | 1.226 |  | 2.328 | 2.063 | 1.888 |  | 2.029 | 1.935 | 1.922 |
| Variance, % | 16.06 | 15.42 | 13.33 | 9.18 |  | 19.40 | 16.18 | 15.73 |  | 20.29 | 19.35 | 19.22 |
|  | 52.99% | | | |  | 52.31% | | |  | 58.86% | | |

Abbreviation: GDS geriatric depression scale

**Appendix 1. Consultation form of the applicability of the GDS-15**

Dear experts:

The Hainan Hospital of Chinese PLA General Hospital is carrying out a study on the modification of the 15-item Geriatric Depression Scale (GDS-15) for the oldest-old (80-99 years) and centenarians. As an expert in the field of Geriatric psychology, we sincerely expect your valuable advice regarding the applicability and modification of the GDS-15 for the oldest-old.

Several GDS versions with different combinations of items were used for depression screening for older adults. Considering the varied characteristics and background of the subjects, individual unsuitable items could be deleted for better and more accurate depression screening. Previous studies have also found that compared to the GDS-15, the simplified version of GDS has reliable accuracy of depression screening and is more applicable for the very old persons. When screening for depression in the older adults from different countries, the regional culture, language, as well as the age strata, physical function, lifestyle and living environment of the subjects must be considered. It is also necessary to evaluate the reliability and validity of the overall scale as well the characteristic of each item for the oldest-old populations.

During the face-to-face interview of the China Hainan Centenarian Cohort Study, our research team found that some items relevant to physical function or social activities in the GDS-15 might not be applicable for the oldest-old or centenarians. Therefore, individual item needs to be simplified according to the opinions of expert consultation. Thus, we would like to further seek your professional perspectives on the modification or simplification of the GDS-15. Please score the applicability of 15 items in the following scale: 1 = not applicable, 2 = not really applicable, 3= general, 4 = basically applicable and 5 = applicable. And select 3-10 items which you think can be deleted. Please consider the following issues: Whether the content of the item is suitable for Chinese cultural and the lifestyle and living environment of the oldest-old; Most of the subjects were living in the community with low physical and social activity; Whether the wording of specific items is comprehensible for the older people with low education level.

**Contact information of the project:**

Zhang Chi 13621313592 zhangchi4616@bjhmoh.cn

Yao Yao 15600113336 yaoyao@nsd.pku.edu.cn

**Principle Investigator:** Zhao Yali 15692538761 zhaoyl301@163.com

**Your basic information:**

Name: Professional Title: Unit: Professional field: Working lives:

**Fifteen items of the Geriatric Depression Scale:**

| Item | Contents | Applicability score | | | | | Select 3-10 items that could be deleted | |
| --- | --- | --- | --- | --- | --- | --- | --- | --- |
|  |  | Not applicable | Not really applicable | General | Basically applicable | Applicable |  |  |
| 1 | Are you generally satisfied with your life? |  |  |  |  |  | Yes | No |
| 2 | Have you given up many of your old activities and hobbies? |  |  |  |  |  | Yes | No |
| 3 | Do you find life boring or empty? |  |  |  |  |  | Yes | No |
| 4 | Do you often feel bored? |  |  |  |  |  | Yes | No |
| 5 | Are you in good spirits most of the time? |  |  |  |  |  | Yes | No |
| 6 | Are you afraid something bad is going to happen to you? |  |  |  |  |  | Yes | No |
| 7 | Are you happy most of the time? |  |  |  |  |  | Yes | No |
| 8 | Do you ever feel like no one is helping you? |  |  |  |  |  | Yes | No |
| 9 | Do you prefer to stay at home, rather than going out? |  |  |  |  |  | Yes | No |
| 10 | Do you feel you have more problems with memory than most? |  |  |  |  |  | Yes | No |
| 11 | Do you think it is wonderful to be alive now? |  |  |  |  |  | Yes | No |
| 12 | Do you feel worthless? |  |  |  |  |  | Yes | No |
| 13 | Do you feel full of energy? |  |  |  |  |  | Yes | No |
| 14 | Do you feel that your situation is hopeless? |  |  |  |  |  | Yes | No |
| 15 | Do you think that most people are better off than you are? |  |  |  |  |  | Yes | No |
|  | | | | | | | | |
| Other comments or suggestions： | | | | | | | | |

**Appendix 2. Information of 19 experts in the consultation**

| Number | Gender | Professional field | Working lives (year) | Affiliated units |
| --- | --- | --- | --- | --- |
| 1 | Male | Geriatrics | 35 | Beijing Hospital; National Center of Gerontology |
| 2 | Male | Neurology; Geriatric psychology | 35 | Beijing Hospital; National Center of Gerontology |
| 3 | Male | Geriatric psychology | 15 | Department of Psychology, Beijing Normal University |
| 4 | Female | Geriatric psychology | 24 | Department of Psychology, Beijing Normal University |
| 5 | Female | Geriatric epidemiology | 21 | Beijing Hospital; National Center of Gerontology |
| 6 | Male | Neurology; Geriatric psychology | 31 | Beijing Hospital; National Center of Gerontology |
| 7 | Male | Neurology | 30 | Second Medical Center of Chinese PLA General Hospital |
| 8 | Female | Psychology | 30 | Department of Medical Psychology, PLA General Hospital |
| 9 | Male | Psychological measurement | 13 | Department of Psychology, School of Sociology, Nanjing University |
| 10 | Female | Geriatrics | 37 | Beijing Hospital; National Center of Gerontology |
| 11 | Female | Applied psychology | 17 | Second Medical Center of Chinese PLA General Hospital |
| 12 | Male | Geriatric psychology | 25 | Hainan Hospital of Chinese PLA General Hospital |
| 13 | Female | Geriatric psychology | 12 | Institute of Psychology, Chinese Academy of Sciences |
| 14 | Female | Geriatric psychiatry | 27 | Peking University Sixth Hospital; Peking University Institute of Mental Health; National Clinical Research Center for Mental Disorders |
| 15 | Male | Epidemiology and psychiatry | 13 | School of Public Health, Guangdong Medical University |
| 16 | Male | Geriatric psychology | 26 | Institute of Psychology, Chinese Academy of Sciences |
| 17 | Male | Geriatric psychology | 10 | Institute of Psychology, Chinese Academy of Sciences |
| 18 | Female | Geriatric psychology | 10 | Karolinska University |
| 19 | Female | Geriatric Cognition and Nursing | 34 | School of Nursing, Central South University |
